# Detecting Suicide and Self-Harm Discussions Among Opioid Substance Users on Instagram Using Machine Learning

**DOI:** 10.3389/fpsyt.2021.551296

**Published:** 2021-05-31

**Authors:** Vidya Purushothaman, Jiawei Li, Tim K. Mackey

**Affiliations:** ^1^Masters in Public Health Program, University of California, San Diego, San Diego, CA, United States; ^2^Department of Anesthesiology, School of Medicine, University of California, San Diego, San Diego, CA, United States; ^3^Department of Healthcare Research and Policy, University of California San Diego - Extension, San Diego, CA, United States; ^4^Global Health Policy and Data Institute, San Diego, CA, United States; ^5^Division of Infectious Disease and Global Public Health, Department of Medicine, School of Medicine, University of California, San Diego, San Diego, CA, United States

**Keywords:** suicide, substance abuse, social media, Instagram, opioids, machine learning

## Abstract

**Background:** Suicide and substance use disorder (SUD) pose serious public health challenges among young adults in the United States. Increasing social media use among these populations can be leveraged as an alternative method to detect characteristics of suicide-related topics and behavior among substance users.

**Objective:** To detect and characterize suicide and self-harm related conversations co-occurring with SUD posts and comments on the popular social media platform Instagram.

**Methods:** This study used big data and machine learning approaches to collect and classify Instagram posts containing 632 controlled substance-related hashtags. Posts were first classified for online drug diversion topics and then filtered to detect suicide and mental health discussions. Posts and comments were then manually annotated for SUD and mental health co-occurring themes. Associations between these characteristics were tested using the Chi-square test.

**Results:** We detected 719 Instagram posts/comments that included user-generated discussions about suicide, substance use and/or mental health. Posts self-reporting SUD and mental health topics were also more likely to discuss suicide compared to those that did not discuss SUD and mental health topics, respectively (*p* < 0.001). Major themes observed included concurrent discussions of suicide ideation and attempts and low self-esteem.

**Conclusions:** Our study results provide preliminary evidence of social media discussions about suicide and mental health among those with SUD. This co-occurrence represents a key health risk factor on a platform heavily utilized by young adults. Further studies are required to analyze specific patterns of suicide and self-harm ideations for the purposes of designing future suicide prevention campaigns through digital channels.

## Introduction

The recent National Vital Statistics Reports (1) has ranked suicide as the second most common cause of death in 10–24 year olds, equating to 47,173 deaths in 2017 (1). In this age group, young adults (18–25 years old) had the highest prevalence of suicide attempts and have also been identified as the largest group of prescription opioid abusers (2). The human toll of the opioid epidemic is also apparent, with more than 70,000 deaths due to drug overdose reported in the U.S. in 2017, out of which more than 65% were related to opioids (3). Importantly, the probability of suicide ideation and attempts were higher in those with increased prescription opioid misuse (4).

Substance use tends to coexist with mental health illnesses and different co-occurrence of mental health issues. For example, individuals attempting suicide can have past or current experiences with substance use disorder (SUD) while also suffering from underlying mental health problems. This includes important interaction points between SUD and mental health status, such as experiencing depression following a failed attempt at successful drug treatment or going through withdrawal (5, 6). In fact, prior studies have reported a 2–3-fold higher risk of suicide associated with substance use among populations who also have mental health illnesses (7). Non-suicidal self-harm, cannabis use, and other illicit substance use have also been reported as the strongest predictors behind the transition from suicide/self-harm ideation to suicide attempts (8).

Concomitantly, according to the Pew Research Center, social media use is widespread among young adults, including for the popular picture and video sharing site Instagram owned by Facebook (67% of 18- to 29-year olds report use of Instagram) (9). These social networking sites have emerged as platforms for sharing thoughts, beliefs or behaviors related to both suicide ideation and SUD, with several high-profile cases leading Instagram to implement reporting tools for content related to suicide or self-injury (10). This includes reports of a Malaysian teen attempting suicide after posting a poll on Instagram of whether to choose life or death (69% of respondents suggested death) and the case of 14-year-old Molly Russell from the United Kingdom who attempted suicide after viewing content about suicide on Instagram in 2017 (10, 11). In response to these events, Instagram has pledged to remove images and drawings related to suicide from its platform and blocks searches for certain suicide-related hashtags (12).

Despite growing debate regarding suicide and self-harm activities posted to social media, young adults also use these platforms to self-report other mental health problems such as depression, anxiety, and bipolar disorder, separate from suicide and self-harm discussions, while also discussing SUD topics, such as opioid abuse (13). This user-generated content can help researchers identify latent and emerging behavioral themes at the intersection of SUD and mental health, which may not otherwise be detectable or reported in a timely manner through other survey instruments. In response, this study aims to detect and characterize suicide and self-harm related conversations associated with opioid discussion among Instagram users using big data and machine learning to better understand the interaction between these two crucial public health challenges.

## Methods

This study was conducted in three phases: (1) data collection; (2) supervised machine learning classification; and (3) text filtering and manual content analysis. Each phase is described below.

### Data Collection

A web scraper was developed in the Python programming language to collect data from the Instagram platform. Instagram posts were then filtered for 632 hashtags related to opioids and other controlled substances with posts collected between July–October 2018. Data was collected as part of a prior published study examining illicit online drug diversion and dealing (14). This dataset yielded user-generated social media conversations about SUD (e.g., users seeking access to drugs, discussing experiences with drug use, etc.) and posts containing illegal drug dealing and selling activities. The original purpose of the published study was to identify and characterize illicit sellers of opioids, other controlled substances, and illicit drugs, but also contained SUD behavioral-related posts and comments of interest. These posts and comments were used in this study for the purpose of identifying and characterizing topics related to SUD and mental health. Data cleaning and processing of our dataset included removing duplicate results of posts and eliminating posts and comments with special characters (except # which represents hashtags), hyperlinks and stop words.

### Supervised Machine Learning

The prior study's dataset that was used in this study employed a supervised machine learning classification approach to detect Instagram posts associated with illegal opioid drug selling and diversion. Specifically, the study used the package Pytorch in Python to develop a deep learning algorithm deploying Long-Short Term Memory (LSTM) and achieved a high accuracy of classification for the detection of illicit drug sellers (i.e., user accounts offering the direct sale of a controlled substance to consumers) (14). A manually annotated set of data was divided into a training set and a validation set to evaluate the performance of the deep learning model along with three other supervised machine learning models. The deep learning model yielded the highest performance among the models evaluated, resulting in a final dataset of 1,228 posts that were confirmed as involved in illegal online drug selling (14). The area under the curve (AUC) was calculated for: (i) text with hashtags; (ii) hashtags only; and (iii) text without hashtags in order to evaluate model performance. The highest AUC was observed for texts without hashtags (99.12%) followed by text with hashtags (98.12%) and only hashtags (94.32%) (14). These posts classified by our deep learning model formed the basis for further exploration of mental health and suicide topics that might be co-occurring in this population of drug sellers and the users they interact with per the aims of this study.

### Keyword Filtering and Content Analysis

Based on the machine learning approach described above, all posts and comments interacting with user accounts identified as engaged in illegal online drug selling were further filtered for specific keywords associated with suicide including: “suicide,” “suicides,” “#suicide.” After this additional keyword filter was applied, all posts and comments were then manually annotated by first and second authors based on the descriptive language, hashtags and/or images contained in the post or comment. An inductive coding scheme was used for content analysis and also served as our inclusion and exclusion criteria. The coding scheme included a binary classification of whether the post/comment discussed self-reported substance use behavior, suicide and/or self-harm or other mental health discussions (see Supplementary Material for detailed description of coding schema). The posts were also manually annotated for specific co-occurring SUD and mental health themes. For inconsistent results, authors reviewed and conferred on the correct classification. Content analysis was followed by Chi-square tests to examine if the proportion of posts containing suicide discussions varied among: (i) substance use related and non-substance use related posts; and (ii) mental health related and non-mental health related posts. Statistical analysis was conducted using Rstudio version 3.6.1 and a *p*-value of < 0.05 was considered statistically significant.

## Results

A total of 56,464 Instagram posts and comments were collected for analysis over a 4-month period from July 2018 to October 2018. Using the machine learning approach described above, 1,228 illegal drug diversion posts were first identified. Further text filtering for suicide-related keywords/hashtags yielded 719 posts that were characterized using manual annotation for the following primary study areas of interest: (1) self-reported substance use behavior; (2) suicide and/or self-harm; and (3) other mental health discussions. The posts were also annotated for discrete as well as concurrent conversations (i.e., substance use in combination with suicide, substance use in combination with mental health discussions, suicide in combination with mental health discussions). Forty-three percent (*n* = 315) were confirmed as “signal” content (i.e., related to at least one study area of interest) with 107 posts 34% discussing suicide either discretely (see Table 1) or concurrently with substance use or other mental health discussions (see Table 2).

**Table 1:** Number of posts related to substance use behavior, suicide and/or self-harm, other mental health discussions (text of posts have been modified to de-identify from user, no user information reported).

**Theme^**a**^**	**Posts^**b**^**	**Example conversation^**c**^**
Suicide/self-harm	107 (34.0)	“*i was attempting to die last night lived through suicide last night”*
Substance Use	89 (28.3)	“*I can't hold on till summer so that I can get fked up and do cocaine or whatever 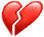 #antixanax #lilxan #xanarchy #diegoleanos #heartbreaksoldier #cocaine @xanxiety”*
Mental health discussion	119 (37.8)	“*I want to kill myself . . . . . . . depression#destroying #anxiety #suicide #messedup #sorry #acting #crying #world #hate #notfine #tired #left #broken #falling #self-harm #neverenough #scars #empty #unloved #imperfections #drug #sober #recovery #disorder #smoking #drunk #ciggarettes”*

a *Discrete or concurrent signal*.

b *Number of posts and the percentage of total signal posts that contained the theme*.

c *Instagram posts/comments with signal*.

**Table 2:** Number of posts with co-occurring discussions related to substance use behavior, suicide and/or self-harm, other mental health discussions (text of posts have been modified to de-identify from user, no user information reported).

**Theme^**a**^**	**Posts^**b**^**	**Example conversation^**c**^**
Suicide concurrent with substance use	52 (16.5)	“*I'm trying to get the most high I can get before I overdose and stop living . . . . #polishgirl #pills #drugaddict #drugs #xanax #xanaxfamily #death #sad #sadgirl #depression #sadness #alone #dank #perscriptiondrugs #junkie #addicted #suicide #suicidal #aesthetic #psycho #goth #emo #killme #ftp #satan #hell #praisethedevil”*
Suicide concurrent with mental health discussion	66 (21.0)	“*I had another difficlut night, Panic Attack, Depression, Tinnitus so loud!, veins in front of face hurt me so much, Anguish is rip me a part, Nightmare on my parents, Screaming out, little Seizure! Singing in Dreams, Heartbeat so faster, Anxiety so back! As Stress!! Suicide mode, tears out but stuck it! And I am stuck at house with not having courage to get out of my room, Almost seven months out of Drugs such as Xanax, and 14 months out of Zyprexa, and yet this sickness, like being the first months out of Drugs! And not seem Improvements, seem not possible to get back to normal life, maybe I didn't having one. So gotta live with or Drugs or gotta Die this winter? Why can see the the light of this Tunnel? I love u, but pain is so much that I to do something, Drugs? Or Death? And not see others nice”*
Substance use concurrent with mental health discussion	43 (13.7)	“*I took 17 different psychiatric drugs over 25 years and life sucked too much/ ignorance is bliss? Not so. But I believed the doctors knew better. I got off all those meds last August and now use only weed as medicine. Sooooo much cheerful and healthier as a consequence and greatly lowered my risk of Alzheimer's dementia depression suicide impotence oversleeping weight gain lethargy. #bigpharmakills #bigpharmasucks #psychmedskill #antidepressants #zoloft #zyprexa #adderrall #ativan #wellbutrin #klonopin #weed #marijuana #cannabisismedicine #weedismedicine #psychiatry”*

a *Discrete or concurrent signal*.

b *Number of posts and the percentage of total signal posts that contained the theme*.

c *Instagram posts/comments with signal*.

**Figure 1:**
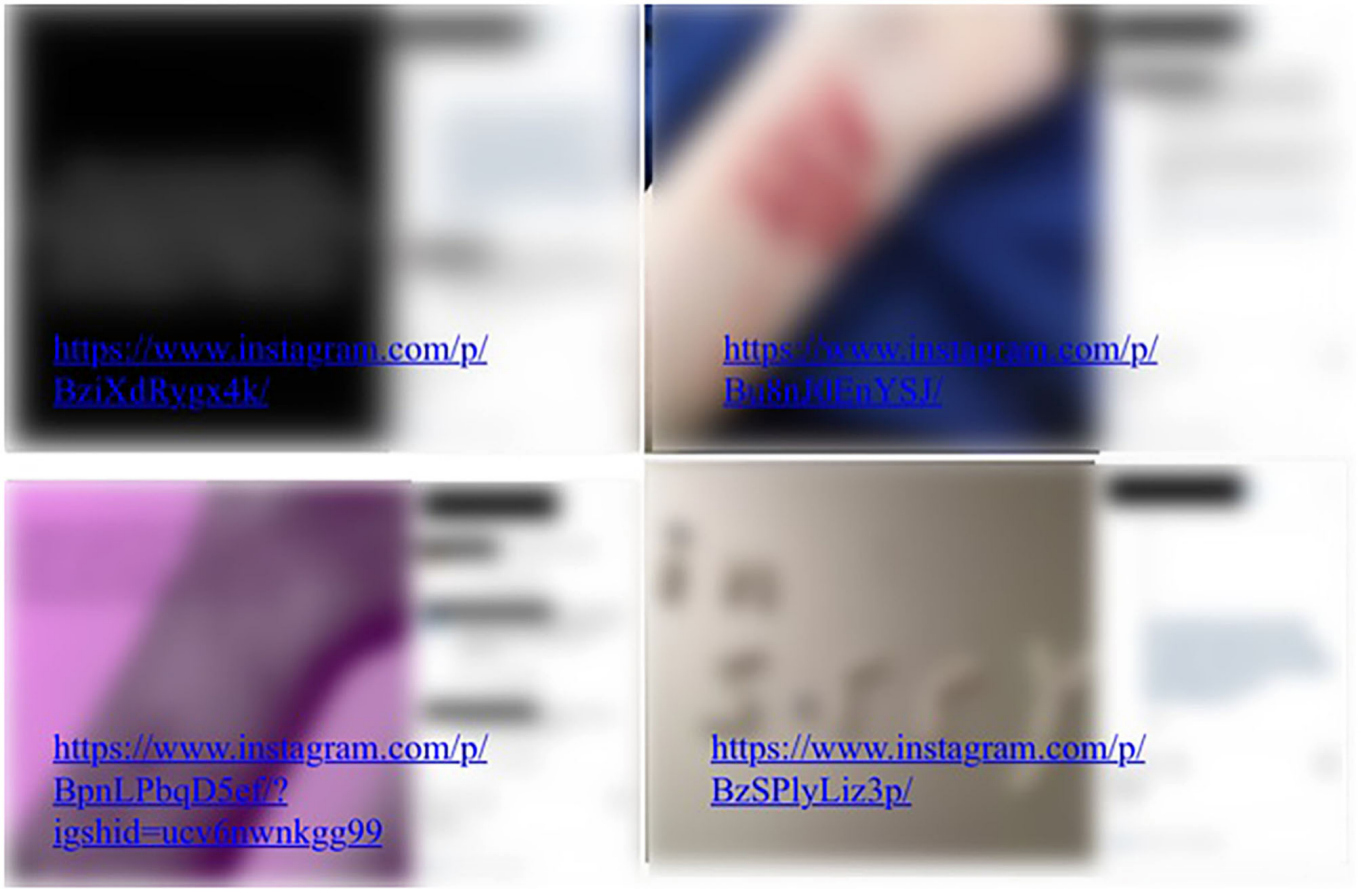
Instagram posts with signal for suicide/self-harm, mental health problems and drug abuse (user information concealed and images blurred). If you're thinking about suicide, are worried about a friend or loved one, or would like emotional support, please contact the National Suicide Prevention Lifeline at 1-800-273-TALK(8255). Available 24 h (https://suicidepreventionlifeline.org/).

In relation to concurrent substance use, suicide and mental health discussion, 52 messages (16.5%) discussed suicide and substance use, 66 messages (21%) included suicide co-occurring with mental health, and 43 messages (13.7%) had simultaneous discussion of substance use and other mental health-related problems (see Figure 1 for examples). Thirty-two messages (10.2%) discussed suicide, substance use and mental health problems concurrently. A chi-square-test of independence showed that there was a significant association between (a) suicide and substance use; and (b) suicide and mental health discussions. Posts containing signal for substance use were more likely to have positive signal for suicide compared to non-substance use related posts, *X*^2^ (1, *N* = 660) = 131.39, *p* < 0.001. Posts containing signal for mental health discussions were more likely to have positive signal for suicide compared to non-mental health related posts, *X*^2^ (1, *N* = 660) = 161.14, *p* < 0.001.

The common prevailing themes that were observed based on the text of the user-generated posts/comments included concurrent discussion of the struggles of addiction leading to mental health challenges, self-harm or suicide ideation with substance use mention along with discussion about depression, concerns regarding lack of substance use treatment efficacy, low self-esteem, and lack of needed social support for mental health care. Posts specific to signal for mental health problems most commonly discussed depression, anxiety and panic attacks with the majority of posts discussing depression along with anxiety. The majority of the signal related to discrete posts on suicide included self-reported attempts of suicide or self-harm or ideations toward the same. Posts with concurrent signal for suicide and substance use discussed intended as well as inadvertent overdosing along with suicide ideation. Posts with concurrent signal for suicide and mental health problems revolved around the social stigma associated with mental health and a lack of peer support leading to a feeling of hopelessness and suicide ideations. Other concurrent discussions on substance use and mental health problems reflected a vague dependence on substance use and lack of belief in seeking professional help for mental health problems.

## Discussion

The results of this study serve as a preliminary evidence about the existing themes around suicide/self-harm ideations concurrent with substance use and mental health illnesses as expressed by users on Instagram, an extremely popular social media platform with a large population of young adult users. While preliminary, the study evidences interaction of these themes in the digital community of Instagram users, adding important insight to current lack of robust data on the interaction between SUD behavior and suicide ideation, which can serve as a starting point in developing surveillance and prevention approaches that more specifically address this co-occurrence. Evidencing the lack of available data on suicide, the World Health Organization reports good quality vital registration data in only 80 member states that can be used to assess suicide rates (15). Hence, innovative surveillance strategies are important to augment existing data and monitor suicide and suicide/self-harm attempts, including methods leveraging technology, “infoveillance,” and similar syndromic surveillance approaches (16). Specifically, data mining of self-disclosed conversations on social media can help in understanding the relationship between suicide and SUD that is not apparent in data available from traditional survey and national health registries.

However, rising social media interaction and exposure to information and peers relating to SUD, suicide, and other mental health issues remains an understudied topic. Other studies found social media advertisements on sites such as Instagram and Twitter increase the odds of teens being exposed to drug use and thereby influence risky behaviors such as self-harm and suicide ideations (17). The preliminary findings of this study indicate that concurrent discussion of substance use and suicide or mental health problems are taking place on Instagram, despite the platform's pledges to remove such content in the wake of real-world tragedies involving its users. Though a controversial topic, conversations on social media sites can also aid in monitoring and identifying suicide among high-risk groups on a larger scale when combined with machine learning approaches. Further, the underlying characteristics of social media conversations on suicide ideation and SUD can be translated into targeted offline and online suicide prevention strategies that are more nuanced to SUD population and those engaged in online activity. Identifying co-occurrence of substance use disorders, suicide/self-harm and mental health problems can also help in designing future prevention tools that can be targeted for addressing co-morbidities of SUD and suicide simultaneously, while also stratified for the needs of different user populations. Other studies have provided evidence of this potential, with social media serving as a potential platform for designing suicide prevention strategies (18) and using social media data to help develop mobile health technology interventions for suicide, both approaches that can potentially benefit from our study results (19).

## Limitations

This study has some limitations. The results of this study are not generalizable as the user characteristics have not been defined and the data collection was limited to a short period of four months (July–October 2018). Although the results reflect upon a specific set of social media users associated with drug diversion posts on Instagram, lack of demographic data restricts their generalizability. User privacy and confidentiality issues also make any risk estimation difficult. The drug dealer data collected and analyzed in the 4-month study period were manually annotated to verify validity of results and model performance. This time lag between data collection and manual annotation may have resulted in deletion of some posts by users relevant to this study. Since text filtering was used on the drug diversion posts that were manually annotated, any discussions about suicide and mental health in the deleted posts could have been inadvertently lost. Further, social media data can rapidly change due to potential platform policy changes, including blocking or preventing searches of suicide-related posts, which can also coincide with changes in a user's real-world environment, such as growing concerns about a rise in substance abuse and mental health issues due to the COVID-19 pandemic (20). Future studies should be designed such that data collection and analysis is closer to real-time in order to better identify emerging trends and enable data-driven substance abuse and suicide prevention programs. It is also important to recognize that there are a wide range of mental health problems with diverse clinical presentations. For the purposes of this study, these conditions were grouped under an overall mental health category partially due to user description of multiple mental health challenges and also to emphasize the co-occurrence of substance use disorder, suicide and overall mental health problems. This study merely identifies the co-occurrence, but further research is needed to better characterize and disentangle the complexity of these co-morbid conditions and their interactions in the context of social media discussions. Also, this study was unable to validate the results using a prospective data sample. Future research should explore the use of prospective samples of social media users associated with opioid use disorder in order to identify and validate progression of possible suicide/self-harm attempts or other co-occurrent mental health issues. Future studies are needed to better elucidate the broader trends, characteristics and scope of discussions converging around SUD, suicide and mental health in this specific population of social media users as well as measuring user reactions and sentiment to these conversations. Also, recognizing patterns of suicide/self-harm behavior across different substance use characteristics such as types of drugs used and polydrug use, can provide insights on priority sub-groups within substance user communities.

## Conclusion

In addition to the importance of these findings in the context of infoveillance for suicide and SUD, it will be critical to identify an ethical means of utilizing these results for targeted prevention and treatment for this at-risk population. Media-based messages targeting substance use population should be designed in such a way that they encourage seeking help from health professionals, without the fear of being judged or being stigmatized. In spite of increased number of mass media campaigns for suicide prevention, studies report limited efficacy (21). This may be due to poor adherence in a mistargeted population. For example, a broad suicide prevention campaign which does not address the factors specific to substance use may have low impact. Hence, the results of this study can help focus on the substance use specific risk factors and comorbidities that can be gathered by analyzing unstructured self-reported data communicated on these platforms which are highly populated by youth and young adults, a critical priority population in need of help, empathy, and intervention, particularly in the digital sphere.

## Data Availability Statement

The raw data supporting the conclusions of this article will be made available by the authors, without undue reservation.

## Ethics Statement

All information collected from this study was from the public domain and the study did not involve any interaction with users. User indefinable information was removed from the study results.

## Author Contributions

VP, JL, and TM jointly collected the data, designed the study, conducted the data analyses, and wrote the manuscript. All authors contributed to the formulation, drafting, completion, and approval of the final manuscript.

## Conflict of Interest

JL and TM are employees of the startup company S-3 Research LLC. S-3 Research is a startup funded and currently supported by the National Institutes of Health—National Institute on Drug Abuse through a Small Business Innovation and Research contract for opioid-related social media research and technology commercialization. S-3 Research was not involved in this study. The remaining author declares that the research was conducted in the absence of any commercial or financial relationships that could be construed as a potential conflict of interest.
