# Supplementary material for: Detecting Suicide and Self-Harm Discussions Among Opioid Substance Users on Instagram Using Machine Learning
Source: Front Psychiatry. 2021 May 31;12:551296. doi: 10.3389/fpsyt.2021.551296 (PMC8200460; doi:10.3389/fpsyt.2021.551296)
Supplement: Supplementary file 1 [file Table_1.docx]

**Supplementary file 1: Coding Scheme for Instagram posts and comments**

Manual annotation of posts and comments: The following coding scheme was used for binary classification of whether the post/comments discussed (1) self-reported substance use behavior; (2) suicide and/or self-harm; and (3) other mental health discussions, and co-occurrence of these themes.

| **THEME** | **SIGNAL** | **CODING SCHEME** |
| --- | --- | --- |
| **Substance Use Behavior** | YES | - Mention of specific substance use related behavior along with mention of type of substance used - Discussing attempts to overdose - Reporting current or past substance use experiences - Reporting substance use by self or friends/family/neighbors (mention of relationship, tagging the profile of other users) - Reporting visiting clinic/urgent care/ER for substance use/overdose |
|  | NO | - Discussion about news posts related to substance use - Discussing substance use effects to create awareness - Sarcasm/jokes about substance use - Reporting substance use among celebrities/other public figures |
| **Suicide/Self-harm** | YES | - Reporting past or present attempts to commit suicide or self-harm - Posting suicide/self-harm related ideations - Posts/comments discussing suicide ideation with or without mention of substance use/overdose |
|  | NO | - Posting about celebrities/other public figures attempting/committing suicide - Discussion about suicide prevention helpline or awareness about professional help available for suicide/self-harm ideations - Sarcasm/jokes on suicide ideation/attempts |
| **Other mental health discussions** | YES | - Reporting symptoms related to depression, anxiety and other mental health conditions - Reporting low self-esteem, lack of social support with/without concurrent discussion on substance use and self-harm ideations |
|  | NO | - Reporting news articles about mental health conditions - Posting about mental health awareness and campaigns/helplines for mental health discussions - Sarcasm/jokes about mental health discussions |
